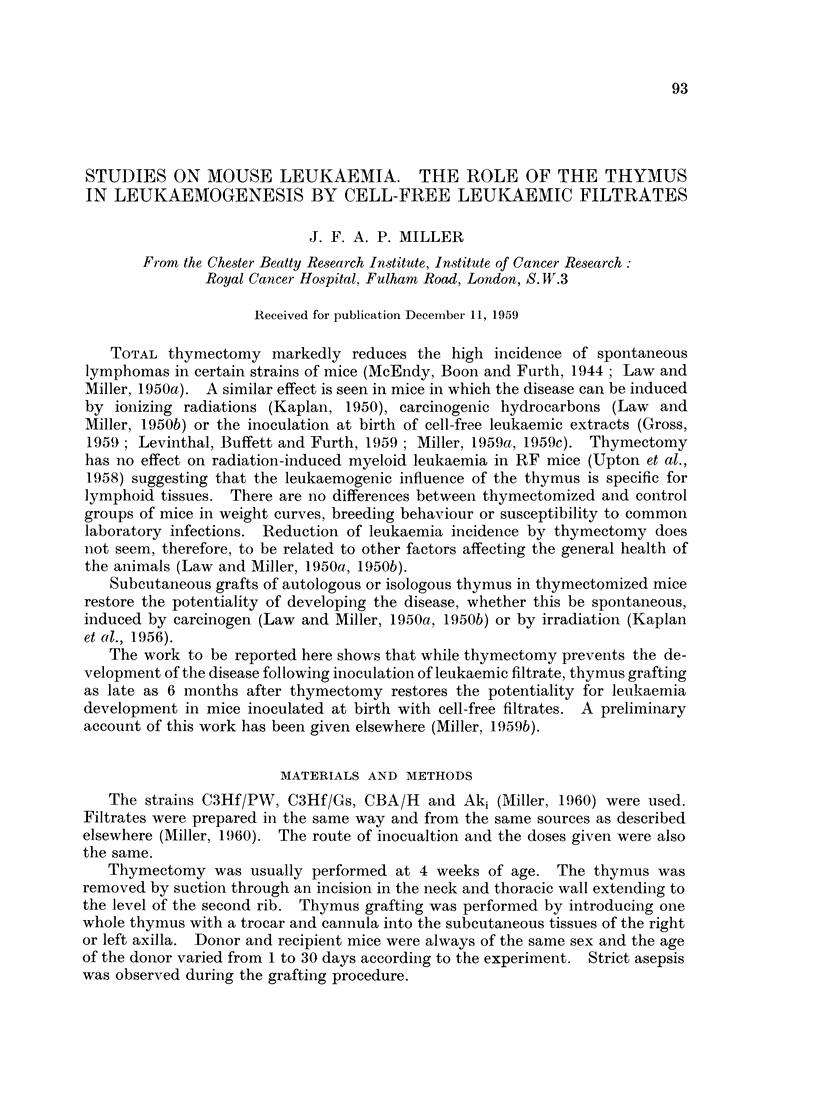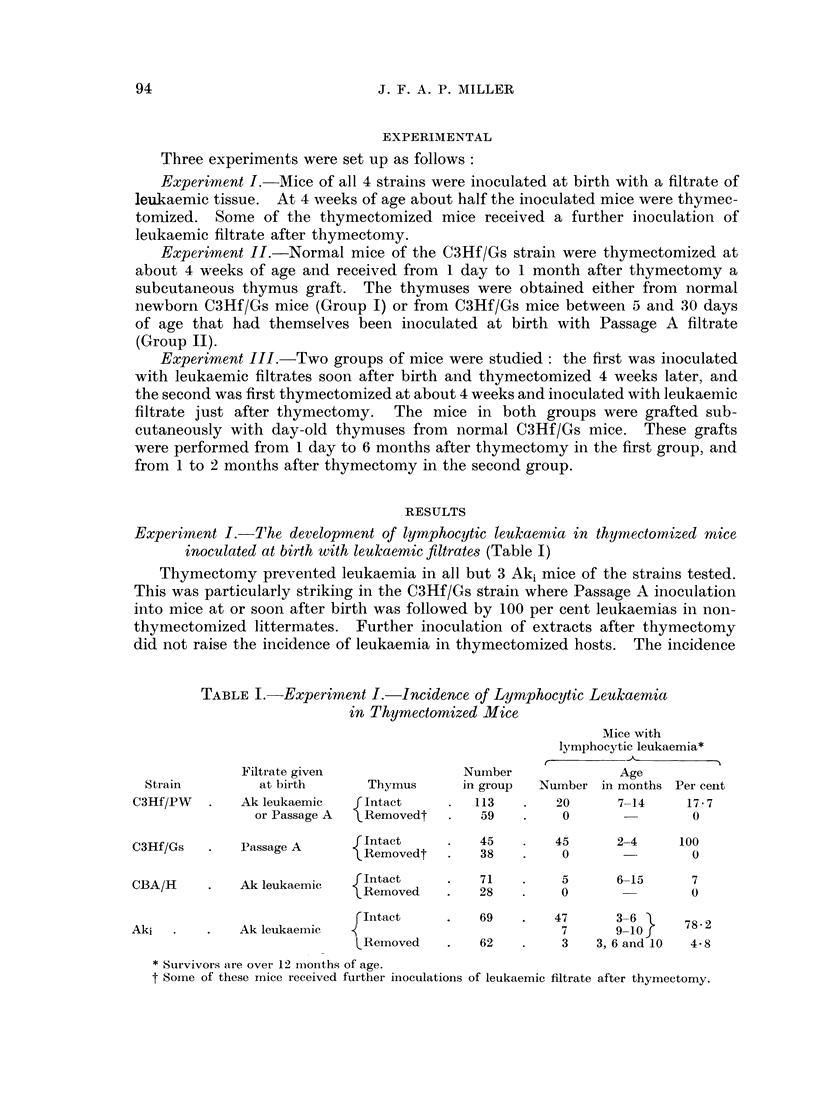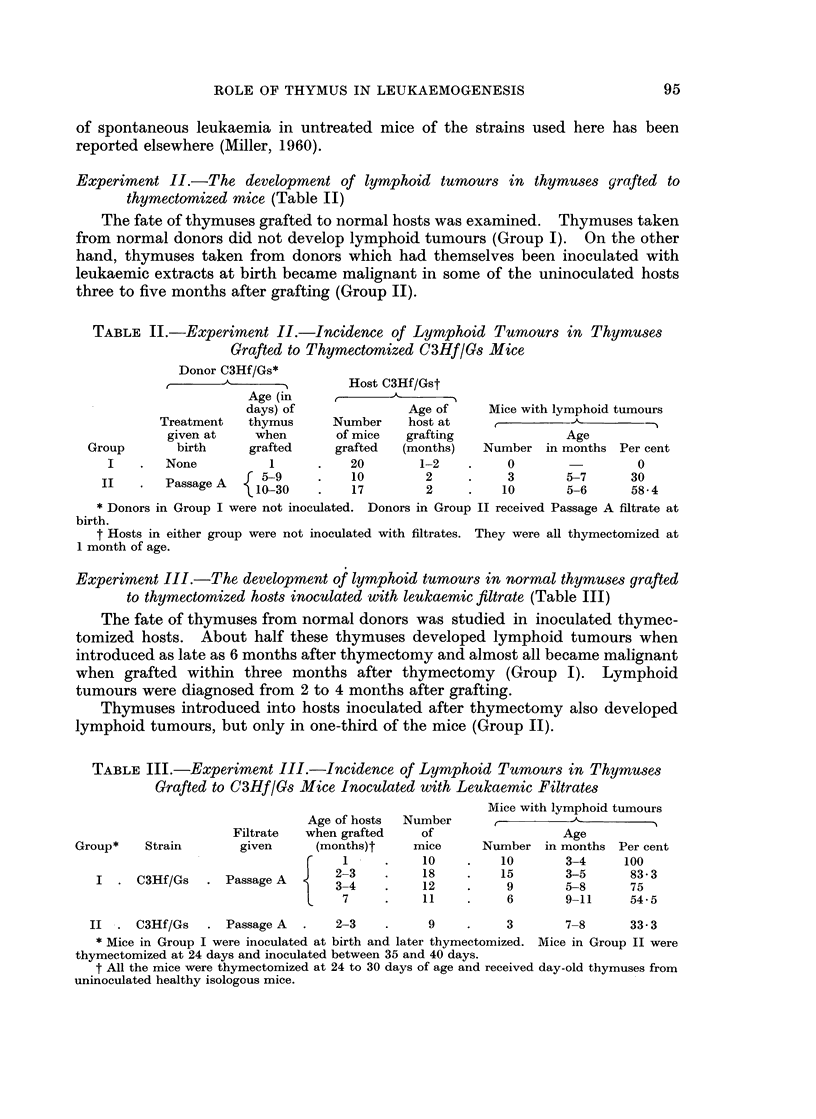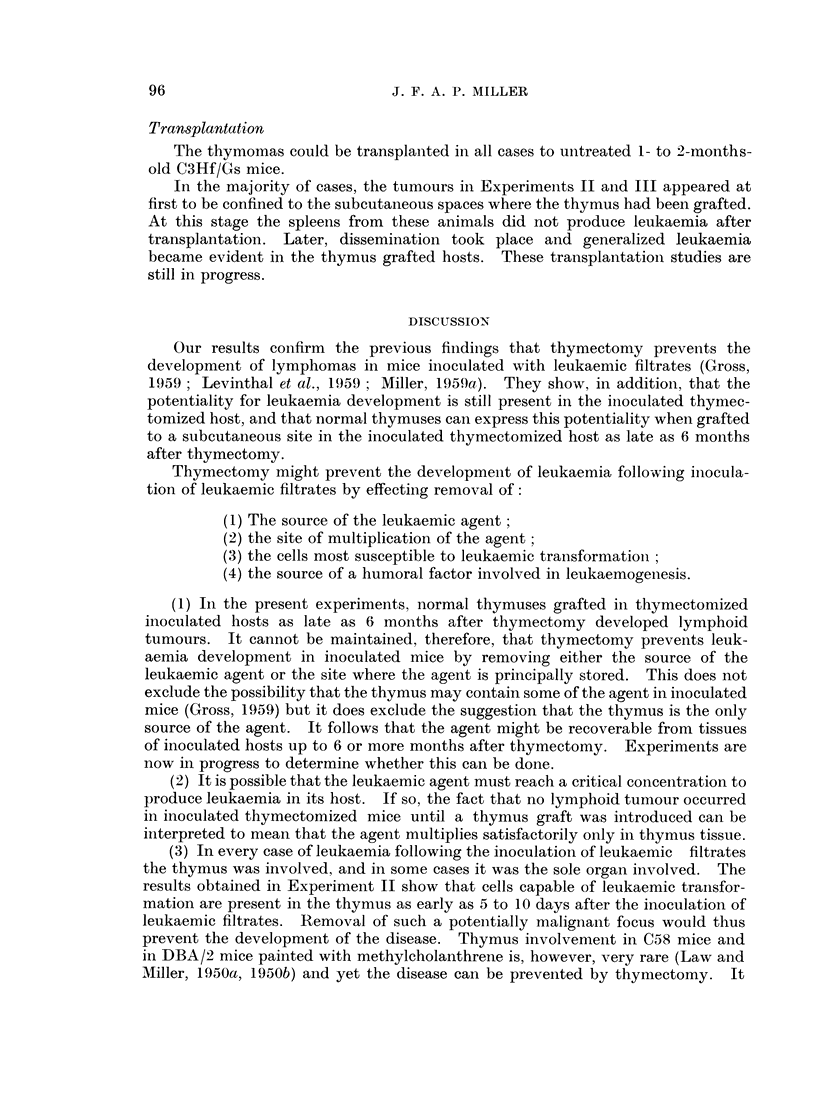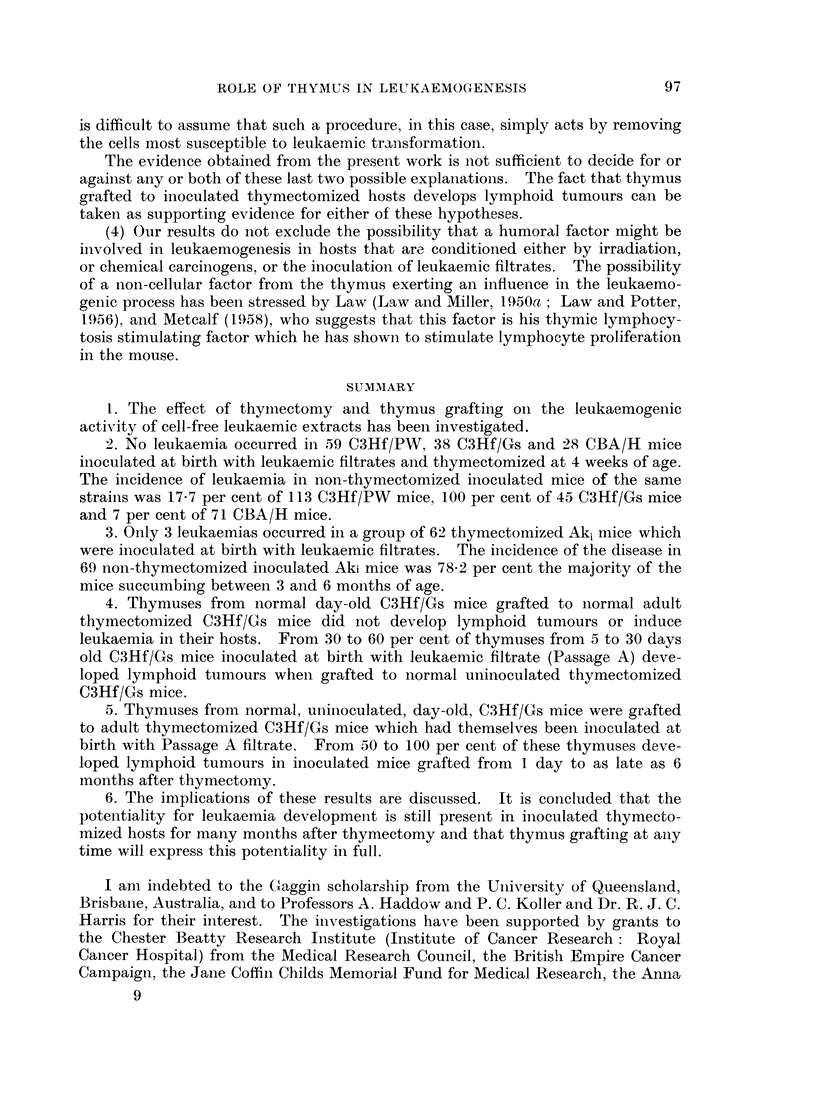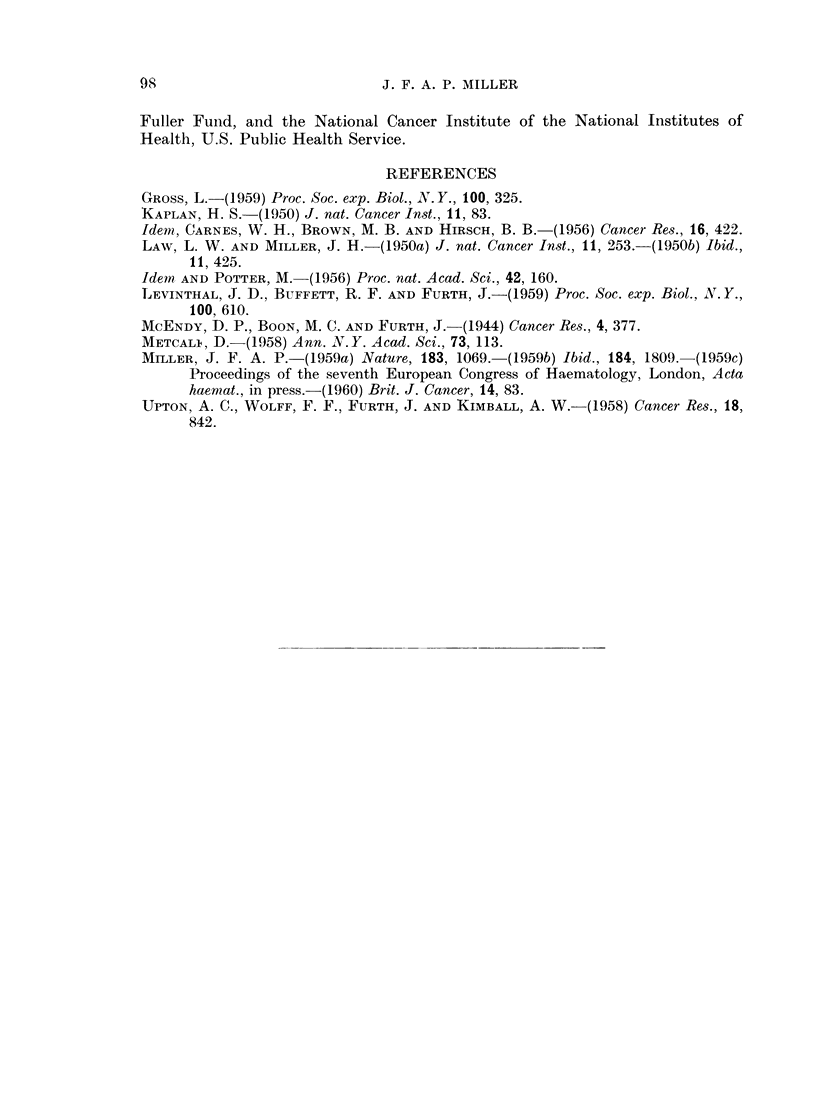# Studies on Mouse Leukaemia. The Role of the Thymus in Leukaemogenesis by Cell-free Leukaemic Filtrates

**DOI:** 10.1038/bjc.1960.11

**Published:** 1960-03

**Authors:** J. F. A. P. Miller


### STUDIES ON MOUSE LEUKAEMIA. THE ROLE OF THE THYMUS IN LEUKAEMOGENESIS BY CELL-FREE LEUKAEMIC FILTRATES

J. F. A. P. MILLER

*From the Chester Beatty Research Institute, Institute of Cancer Research : Royal Cancer Hospital, Fulham Road, London, S.W.3*

Received for publication December 11, 1959

TOTAL thymectomy markedly reduces the high incidence of spontaneous lymphomas in certain strains of mice (McEndy, Boon and Furth, 1944 ; Law and Miller, 1950*a*). A similar effect is seen in mice in which the disease can be induced by ionizing radiations (Kaplan, 1950), carcinogenic hydrocarbons (Law and Miller, 1950*b*) or the inoculation at birth of cell-free leukaemic extracts (Gross, 1959 ; Levinthal, Buffett and Furth, 1959 ; Miller, 1959*a*, 1959*c*). Thymectomy has no effect on radiation-induced myeloid leukaemia in RF mice (Upton *et al.*, 1958) suggesting that the leukaemogenic influence of the thymus is specific for lymphoid tissues. There are no differences between thymectomized and control groups of mice in weight curves, breeding behaviour or susceptibility to common laboratory infections. Reduction of leukaemia incidence by thymectomy does not seem, therefore, to be related to other factors affecting the general health of the animals (Law and Miller, 1950*a*, 1950*b*).

Subcutaneous grafts of autologous or isologous thymus in thymectomized mice restore the potentiality of developing the disease, whether this be spontaneous, induced by carcinogen (Law and Miller, 1950*a*, 1950*b*) or by irradiation (Kaplan *et al.*, 1956).

The work to be reported here shows that while thymectomy prevents the development of the disease following inoculation of leukaemic filtrate, thymus grafting as late as 6 months after thymectomy restores the potentiality for leukaemia development in mice inoculated at birth with cell-free filtrates. A preliminary account of this work has been given elsewhere (Miller, 1959*b*).

### MATERIALS AND METHODS

The strains C3Hf/PW, C3Hf/Gs, CBA/H and $Ak_i$ (Miller, 1960) were used. Filtrates were prepared in the same way and from the same sources as described elsewhere (Miller, 1960). The route of inocualtion and the doses given were also the same.

Thymectomy was usually performed at 4 weeks of age. The thymus was removed by suction through an incision in the neck and thoracic wall extending to the level of the second rib. Thymus grafting was performed by introducing one whole thymus with a trocar and cannula into the subcutaneous tissues of the right or left axilla. Donor and recipient mice were always of the same sex and the age of the donor varied from 1 to 30 days according to the experiment. Strict asepsis was observed during the grafting procedure.

### EXPERIMENTAL

Three experiments were set up as follows :

*Experiment I.*—Mice of all 4 strains were inoculated at birth with a filtrate of leukaemic tissue. At 4 weeks of age about half the inoculated mice were thymectomized. Some of the thymectomized mice received a further inoculation of leukaemic filtrate after thymectomy.

*Experiment II.*—Normal mice of the C3Hf/Gs strain were thymectomized at about 4 weeks of age and received from 1 day to 1 month after thymectomy a subcutaneous thymus graft. The thymuses were obtained either from normal newborn C3Hf/Gs mice (Group I) or from C3Hf/Gs mice between 5 and 30 days of age that had themselves been inoculated at birth with Passage A filtrate (Group II).

*Experiment III.*—Two groups of mice were studied : the first was inoculated with leukaemic filtrates soon after birth and thymectomized 4 weeks later, and the second was first thymectomized at about 4 weeks and inoculated with leukaemic filtrate just after thymectomy. The mice in both groups were grafted subcutaneously with day-old thymuses from normal C3Hf/Gs mice. These grafts were performed from 1 day to 6 months after thymectomy in the first group, and from 1 to 2 months after thymectomy in the second group.

### RESULTS

*Experiment I.—The development of lymphocytic leukaemia in thymectomized mice inoculated at birth with leukaemic filtrates* (Table I)

Thymectomy prevented leukaemia in all but 3 $Ak_i$ mice of the strains tested. This was particularly striking in the C3Hf/Gs strain where Passage A inoculation into mice at or soon after birth was followed by 100 per cent leukaemias in non-thymectomized littermates. Further inoculation of extracts after thymectomy did not raise the incidence of leukaemia in thymectomized hosts. The incidence

TABLE I.—*Experiment I.—Incidence of Lymphocytic Leukaemia in Thymectomized Mice*

| Strain | Filtrate given at birth | Thymus | Number in group | Mice with lymphocytic leukaemia* Number | Age in months | Per cent |
|---|---|---|---|---|---|---|
| C3Hf/PW | Ak leukaemic or Passage A | Intact | 113 | 20 | 7–14 | 17·7 |
| | | Removed† | 59 | 0 | — | 0 |
| C3Hf/Gs | Passage A | Intact | 45 | 45 | 2–4 | 100 |
| | | Removed† | 38 | 0 | — | 0 |
| CBA/H | Ak leukaemic | Intact | 71 | 5 | 6–15 | 7 |
| | | Removed | 28 | 0 | — | 0 |
| $Ak_i$ | Ak leukaemic | Intact | 69 | 47 | 3–6 | 78·2 |
| | | | | 7 | 9–10 | |
| | | Removed | 62 | 3 | 3, 6 and 10 | 4·8 |

* Survivors are over 12 months of age.

† Some of these mice received further inoculations of leukaemic filtrate after thymectomy.

of spontaneous leukaemia in untreated mice of the strains used here has been reported elsewhere (Miller, 1960).

*Experiment II.—The development of lymphoid tumours in thymuses grafted to thymectomized mice* (Table II)

The fate of thymuses grafted to normal hosts was examined. Thymuses taken from normal donors did not develop lymphoid tumours (Group I). On the other hand, thymuses taken from donors which had themselves been inoculated with leukaemic extracts at birth became malignant in some of the uninoculated hosts three to five months after grafting (Group II).

TABLE II.—*Experiment II.—Incidence of Lymphoid Tumours in Thymuses Grafted to Thymectomized C3Hf/Gs Mice*

| Group | Donor C3Hf/Gs* Treatment given at birth | Donor C3Hf/Gs* Age (in days) of thymus when grafted | Host C3Hf/Gs† Number of mice grafted | Host C3Hf/Gs† Age of host at grafting (months) | Mice with lymphoid tumours Number | Mice with lymphoid tumours Age in months | Mice with lymphoid tumours Per cent |
|---|---|---|---|---|---|---|---|
| I | None | 1 | 20 | 1–2 | 0 | — | 0 |
| II | Passage A | 5–9 | 10 | 2 | 3 | 5–7 | 30 |
| | | 10–30 | 17 | 2 | 10 | 5–6 | 58·4 |

* Donors in Group I were not inoculated. Donors in Group II received Passage A filtrate at birth.

† Hosts in either group were not inoculated with filtrates. They were all thymectomized at 1 month of age.

*Experiment III.—The development of lymphoid tumours in normal thymuses grafted to thymectomized hosts inoculated with leukaemic filtrate* (Table III)

The fate of thymuses from normal donors was studied in inoculated thymectomized hosts. About half these thymuses developed lymphoid tumours when introduced as late as 6 months after thymectomy and almost all became malignant when grafted within three months after thymectomy (Group I). Lymphoid tumours were diagnosed from 2 to 4 months after grafting.

Thymuses introduced into hosts inoculated after thymectomy also developed lymphoid tumours, but only in one-third of the mice (Group II).

TABLE III.—*Experiment III.—Incidence of Lymphoid Tumours in Thymuses Grafted to C3Hf/Gs Mice Inoculated with Leukaemic Filtrates*

| Group* | Strain | Filtrate given | Age of hosts when grafted (months)† | Number of mice | Mice with lymphoid tumours Number | Mice with lymphoid tumours Age in months | Mice with lymphoid tumours Per cent |
|---|---|---|---|---|---|---|---|
| I | C3Hf/Gs | Passage A | 1 | 10 | 10 | 3–4 | 100 |
| | | | 2–3 | 18 | 15 | 3–5 | 83·3 |
| | | | 3–4 | 12 | 9 | 5–8 | 75 |
| | | | 7 | 11 | 6 | 9–11 | 54·5 |
| II | C3Hf/Gs | Passage A | 2–3 | 9 | 3 | 7–8 | 33·3 |

* Mice in Group I were inoculated at birth and later thymectomized. Mice in Group II were thymectomized at 24 days and inoculated between 35 and 40 days.

† All the mice were thymectomized at 24 to 30 days of age and received day-old thymuses from uninoculated healthy isologous mice.

*Transplantation*

The thymomas could be transplanted in all cases to untreated 1- to 2-months-old C3Hf/Gs mice.

In the majority of cases, the tumours in Experiments II and III appeared at first to be confined to the subcutaneous spaces where the thymus had been grafted. At this stage the spleens from these animals did not produce leukaemia after transplantation. Later, dissemination took place and generalized leukaemia became evident in the thymus grafted hosts. These transplantation studies are still in progress.

### DISCUSSION

Our results confirm the previous findings that thymectomy prevents the development of lymphomas in mice inoculated with leukaemic filtrates (Gross, 1959 ; Levinthal *et al.*, 1959 ; Miller, 1959*a*). They show, in addition, that the potentiality for leukaemia development is still present in the inoculated thymectomized host, and that normal thymuses can express this potentiality when grafted to a subcutaneous site in the inoculated thymectomized host as late as 6 months after thymectomy.

Thymectomy might prevent the development of leukaemia following inoculation of leukaemic filtrates by effecting removal of :

(1) The source of the leukaemic agent ;
(2) the site of multiplication of the agent ;
(3) the cells most susceptible to leukaemic transformation ;
(4) the source of a humoral factor involved in leukaemogenesis.

(1) In the present experiments, normal thymuses grafted in thymectomized inoculated hosts as late as 6 months after thymectomy developed lymphoid tumours. It cannot be maintained, therefore, that thymectomy prevents leukaemia development in inoculated mice by removing either the source of the leukaemic agent or the site where the agent is principally stored. This does not exclude the possibility that the thymus may contain some of the agent in inoculated mice (Gross, 1959) but it does exclude the suggestion that the thymus is the only source of the agent. It follows that the agent might be recoverable from tissues of inoculated hosts up to 6 or more months after thymectomy. Experiments are now in progress to determine whether this can be done.

(2) It is possible that the leukaemic agent must reach a critical concentration to produce leukaemia in its host. If so, the fact that no lymphoid tumour occurred in inoculated thymectomized mice until a thymus graft was introduced can be interpreted to mean that the agent multiplies satisfactorily only in thymus tissue.

(3) In every case of leukaemia following the inoculation of leukaemic filtrates the thymus was involved, and in some cases it was the sole organ involved. The results obtained in Experiment II show that cells capable of leukaemic transformation are present in the thymus as early as 5 to 10 days after the inoculation of leukaemic filtrates. Removal of such a potentially malignant focus would thus prevent the development of the disease. Thymus involvement in C58 mice and in DBA/2 mice painted with methylcholanthrene is, however, very rare (Law and Miller, 1950*a*, 1950*b*) and yet the disease can be prevented by thymectomy. It is difficult to assume that such a procedure, in this case, simply acts by removing the cells most susceptible to leukaemic transformation.

The evidence obtained from the present work is not sufficient to decide for or against any or both of these last two possible explanations. The fact that thymus grafted to inoculated thymectomized hosts develops lymphoid tumours can be taken as supporting evidence for either of these hypotheses.

(4) Our results do not exclude the possibility that a humoral factor might be involved in leukaemogenesis in hosts that are conditioned either by irradiation, or chemical carcinogens, or the inoculation of leukaemic filtrates. The possibility of a non-cellular factor from the thymus exerting an influence in the leukaemogenic process has been stressed by Law (Law and Miller, 1950*a* ; Law and Potter, 1956), and Metcalf (1958), who suggests that this factor is his thymic lymphocytosis stimulating factor which he has shown to stimulate lymphocyte proliferation in the mouse.

### SUMMARY

1. The effect of thymectomy and thymus grafting on the leukaemogenic activity of cell-free leukaemic extracts has been investigated.

2. No leukaemia occurred in 59 C3Hf/PW, 38 C3Hf/Gs and 28 CBA/H mice inoculated at birth with leukaemic filtrates and thymectomized at 4 weeks of age. The incidence of leukaemia in non-thymectomized inoculated mice of the same strains was 17·7 per cent of 113 C3Hf/PW mice, 100 per cent of 45 C3Hf/Gs mice and 7 per cent of 71 CBA/H mice.

3. Only 3 leukaemias occurred in a group of 62 thymectomized $Ak_i$ mice which were inoculated at birth with leukaemic filtrates. The incidence of the disease in 69 non-thymectomized inoculated $Ak_i$ mice was 78·2 per cent the majority of the mice succumbing between 3 and 6 months of age.

4. Thymuses from normal day-old C3Hf/Gs mice grafted to normal adult thymectomized C3Hf/Gs mice did not develop lymphoid tumours or induce leukaemia in their hosts. From 30 to 60 per cent of thymuses from 5 to 30 days old C3Hf/Gs mice inoculated at birth with leukaemic filtrate (Passage A) developed lymphoid tumours when grafted to normal uninoculated thymectomized C3Hf/Gs mice.

5. Thymuses from normal, uninoculated, day-old, C3Hf/Gs mice were grafted to adult thymectomized C3Hf/Gs mice which had themselves been inoculated at birth with Passage A filtrate. From 50 to 100 per cent of these thymuses developed lymphoid tumours in inoculated mice grafted from 1 day to as late as 6 months after thymectomy.

6. The implications of these results are discussed. It is concluded that the potentiality for leukaemia development is still present in inoculated thymectomized hosts for many months after thymectomy and that thymus grafting at any time will express this potentiality in full.

I am indebted to the Gaggin scholarship from the University of Queensland, Brisbane, Australia, and to Professors A. Haddow and P. C. Koller and Dr. R. J. C. Harris for their interest. The investigations have been supported by grants to the Chester Beatty Research Institute (Institute of Cancer Research : Royal Cancer Hospital) from the Medical Research Council, the British Empire Cancer Campaign, the Jane Coffin Childs Memorial Fund for Medical Research, the Anna

Fuller Fund, and the National Cancer Institute of the National Institutes of Health, U.S. Public Health Service.

### REFERENCES

GROSS, L.—(1959) *Proc. Soc. exp. Biol., N.Y.*, **100**, 325.
KAPLAN, H. S.—(1950) *J. nat. Cancer Inst.*, **11**, 83.
*Idem*, CARNES, W. H., BROWN, M. B. AND HIRSCH, B. B.—(1956) *Cancer Res.*, **16**, 422.
LAW, L. W. AND MILLER, J. H.—(1950*a*) *J. nat. Cancer Inst.*, **11**, 253.—(1950*b*) *Ibid.*, **11**, 425.
*Idem* AND POTTER, M.—(1956) *Proc. nat. Acad. Sci.*, **42**, 160.
LEVINTHAL, J. D., BUFFETT, R. F. AND FURTH, J.—(1959) *Proc. Soc. exp. Biol., N.Y.*, **100**, 610.
MCENDY, D. P., BOON, M. C. AND FURTH, J.—(1944) *Cancer Res.*, **4**, 377.
METCALF, D.—(1958) *Ann. N.Y. Acad. Sci.*, **73**, 113.
MILLER, J. F. A. P.—(1959*a*) *Nature*, **183**, 1069.—(1959*b*) *Ibid.*, **184**, 1809.—(1959*c*) Proceedings of the seventh European Congress of Haematology, London, *Acta haemat.*, in press.—(1960) *Brit. J. Cancer*, **14**, 83.
UPTON, A. C., WOLFF, F. F., FURTH, J. AND KIMBALL, A. W.—(1958) *Cancer Res.*, **18**, 842.